# Wicked Problems, Novel Solutions: Nepalese Elephant Tourism and Conservation

**DOI:** 10.3390/ani14010171

**Published:** 2024-01-04

**Authors:** Michelle Szydlowski

**Affiliations:** St. Luke’s Campus, University of Exeter, Exeter EX12LU, UK; michelle@szyd.me

**Keywords:** anthrozoology, Asian elephant, captive–wild interface, conservation, multispecies ethnography, Nepal, tourism

## Abstract

**Simple Summary:**

The conservation of endangered Asian elephants (especially when driven by those from non-range countries) is made more complex in cultures where ‘owning’ individuals for tourism practice is widely accepted. How can a wide variety of stakeholders find common ground upon which to build conservation plans amidst shifting social and environmental pressures?

**Abstract:**

Endangered Asian elephants (*Elephas maximus*) find themselves at the center of debates involving politics, land use, human–wildlife conflict, and environmental justice. The intensity of such debates has led scholars to label conservation challenges as wicked problems with profound implications on local and global practice. In elephant range states such as Nepal, these debates are made more complex through human ‘ownership’ of endangered individuals for use in tourism, worship, or co-work. Human perspectives on the ethics of using animals for tourism are changing, even in areas heavily reliant on the tourism industry for survival. These debates become inflamed when non-residents take on adversarial positions despite an acceptance of the ‘ownership’ or ‘use’ of endangered individuals among local communities. Novel approaches are needed if there is any hope of establishing a common ground upon which to build relationships which may benefit community members, international interests, and endangered individuals.

## 1. Introduction

During research involving Greater-one Horned rhinoceros (*Rhinoceros unicornis*) conservation in Nepal, I found myself spending large periods of time within the elephant stables surrounding Chitwan National Park. Through chance interactions with the residents of these hattisars (hatti is elephant in Nepali and hattisar is elephant stable), I developed a working relationship with both Asian elephants (*Elephas maximus*) and humans (*Homo sapiens*) (Using scientific nomenclature for both species is important, as it problematizes the human-centered habit of listing the Latin names of only so called ‘other than human’ species [1]). I noticed vast differences in the health, welfare, treatment, and behavior of individual elephants who transported researchers into protected areas, served as mounts for anti-poaching patrols, worked with government employees on conservation efforts and forest management, and those who ferried tourists through the national park’s protected areas. Depending upon their ownership status and physical location, each elephant faced drastically different challenges as well as levels of legislative protection [2,3]; for many, being ‘owned’ by different organizations impacted their physical and mental health as well as their expected life span. Likewise, mahouts experienced difficulties arising from their physical location, with some facing inadequate health care or housing at stable facilities. In addition, mahouts face marginalization from community members’ preconceived notions regarding their choices of profession, caste, or their socioeconomic status [4,5,6].

**Figure 1:**
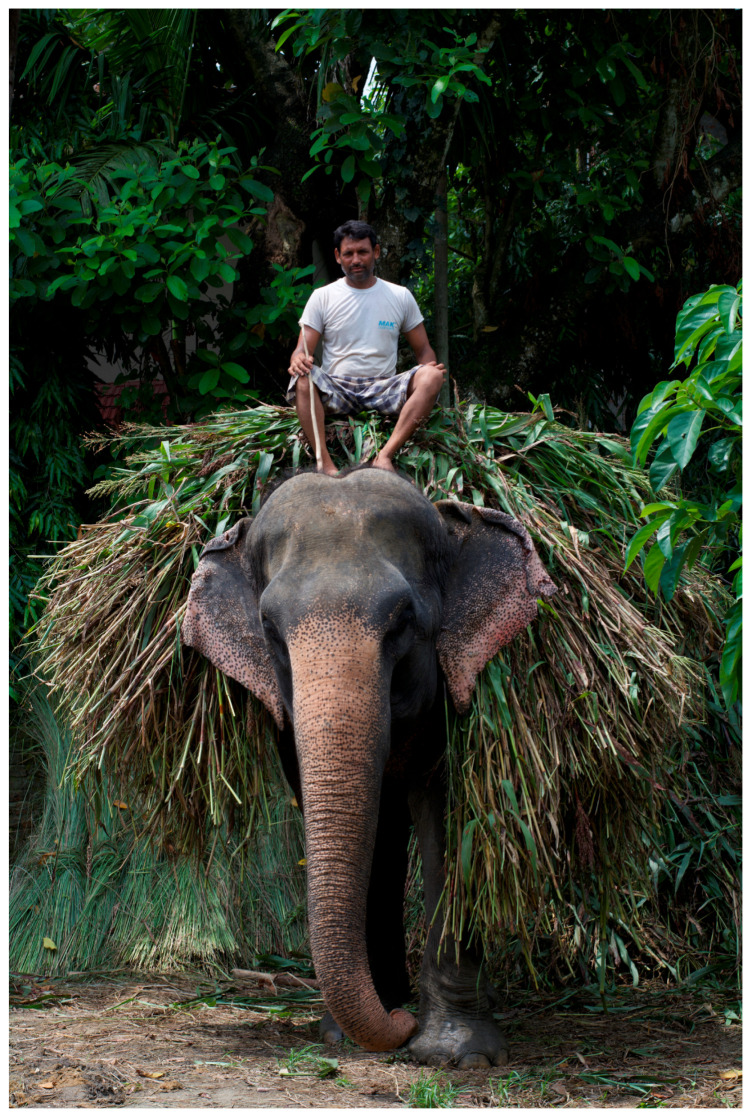
Mahout and elephant co-workers returning home after cutting grass for fodder. Sauraha, Nepal. Photo by Ram Krishna Mahato for this project.

I found myself spending increasing amounts of time examining the reactions of tourists, community members, researchers, and staff from international non-governmental agencies (INGOS) as they visited these elephant–human working pairs, especially those involved in tourism practice (in fact, I ended up revising my doctoral plan and received ethical approval to study human–elephant–NGO relations in Nepal). Both local community members and visitors from other parts of Nepal initially expressed little concern with current elephant-use practices. However, tourists from the Global North, especially those from North America, described themselves as having conflicted or negative perceptions of practices which ‘used’ elephants solely for entertainment purposes [7]. I was initially hesitant to engage in research involving these conflicting attitudes and the challenges faced by tourism elephants in Nepal. This reluctance was due in part to my education as an anthrozoologist (someone who studies human–other-than-human animal relationships), which was steeped in anthropological theory. As such, I understood the need to be reflexive regarding the potential for perpetuating neocolonial attitudes (ideas or beliefs which assume indigenous knowledge is somehow less valuable than that of so-called ‘more-developed’ societies [8]) and was concerned about how to represent elephant–human coworking conditions adequately and fairly. I felt uncomfortable discussing the ownership and treatment of elephants in Nepal through a ‘western’ welfare lens, if, as some alleged, local attitudes were generally supportive of the practice. I admit to experiencing discomfort as feelings of cultural and moral relativism swirled through my head, and I wrestled with conflicting information from elephant owners, mahouts, veterinary staff, biologists, elephant advocates, and other stakeholders. I wrestled with (erroneous) claims by Nepalese interlocuters that elephants had ‘always’ been used in both forest management and tourism [9]. They explained that thanks to such ‘traditional’ practice, the use of elephants for any human purpose was justifiable. This information convinced me, temporarily, that due to millennia of embodied knowledge shared with pachyderms, Nepalese mahouts and elephant owners must know better than anyone how to properly use and care for elephants (see Figure 1). It is important to note that the author is neither endorsing nor vilifying the keeping of captive wildlife, a debate beyond the scope of this short paper.

However, I remained bothered by the number of owners who treated both elephants and mahouts with indifference, neglect, or as ‘property’. In addition, not all mahouts treated their elephant co-workers with kindness, and some neglected or abused elephants with alarming regularity. Of course, many ‘western’ bosses also fail to treat their employees as beings worthy of respect, and many co-workers fail to approach colleagues with appreciation and respect. Likewise, animals who share their lives with humans in the US can be mistreated; captive wildlife in zoos or aquaria have their agency removed to fulfil public desire for entertainment and scientific demand for breeding stock. Humans regularly fail to care for, or directly abuse, the companion animals that share their homes and communities or work alongside law enforcement and military personnel [10,11]. However, these failures to treat all species equitably do not automatically mean that all guardianship of companion animals should be outlawed (I prefer this term over ‘ownership’). I was, at the time of my initial research, misinformed regarding the problematic and complex history surrounding elephant–human relationships in Nepal, and misguided in my comparison between domestic companion animals and captive wildlife. My initial instinct to ‘avoid getting involved’ was both naïve and possibly irresponsible. Using a fear of potential accusations of neocolonialism to talk oneself out of advocating is an ethical slippery slope. But as readers will discover throughout this paper, such naivety turned out to be a beneficial characteristic while researching elephant and human relations within the complex conservation and tourist landscape of Nepal; it allowed the use of novel and creative approaches to wicked conservation problems. According to Game et al. (2013) [12], such novel approaches are key to establishing a common ground upon which to build working relationships which may benefit elephants, advocates, conservationists, and other stakeholders in the area around Chitwan National Park (henceforth CNP).

During fieldwork in 2019, I noticed a sign offering ‘ethical’ elephant activities—a new idea for Sauraha. Out of curiosity and a sense of hopefulness regarding the living situations of both elephants and mahouts, I reached out to the sign’s creators. Within hours, the focus of my research veered directly into the path of the largest animal in Nepal. As it turned out, numerous local community organizations, including the nature guide association, local businesses, and school groups, were advocating to cease elephant-backed safari. In addition, several international NGOs had entered the area. Due to these changing attitudes, it seemed the time had come for a study of the ethics and care of captive elephants in Sauraha, and I redirected my research into Nepal’s hattisars. However, undertaking this type of study meant that I would have to spend time observing elephants and mahouts as they were mistreated and marginalized. It also meant attempting to incorporate views from a wide variety of individuals: indigenous and international experts, local and foreign veterinary staff, advocates, and welfare specialists from a variety of cultures. It meant trying to equally represent each stakeholder’s truth of value laden words such as ‘care’, ‘welfare’, and ‘conservation’. In other words, it required novel solutions of the kind Game et al. [12] described above. This paper discusses the complex views of stakeholders in Nepal and the ongoing struggles between researchers, owners, advocates, elephants, and governments. It also describes the wicked problems faced by those purporting to practice endangered species conservation. Lastly, it discusses novel approaches to unifying efforts towards improving elephant health.

Information in the following article was derived, in part, from participant observations and interviews with mahouts, owners, I/NGO staff, community members and advocates in ongoing dialogues between 2014 and 2022 in person, via email and messaging apps. These interviews are simply noted as PC (personal communication).

## 2. Wicked Problem One: Conserving Wild Elephants

**Figure 2:**
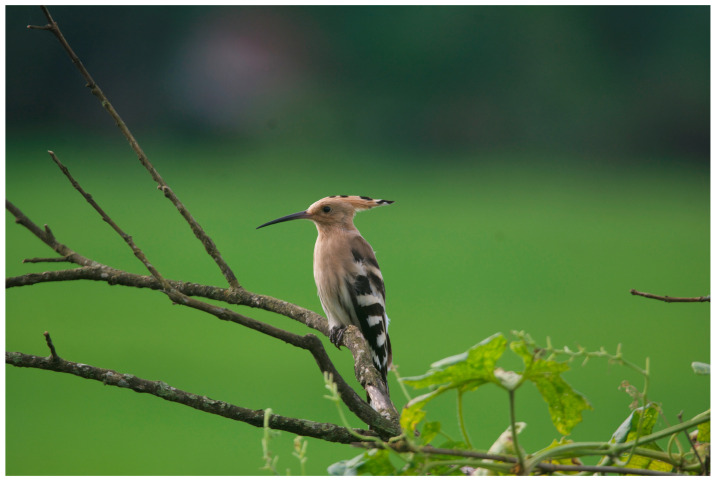
Hoopoe (*Upupa epops*). Chitwan National Park (henceforth CNP), Nepal. Photos by Nepal Dynamic Eco Tours, used with permission.

**Figure 3:**
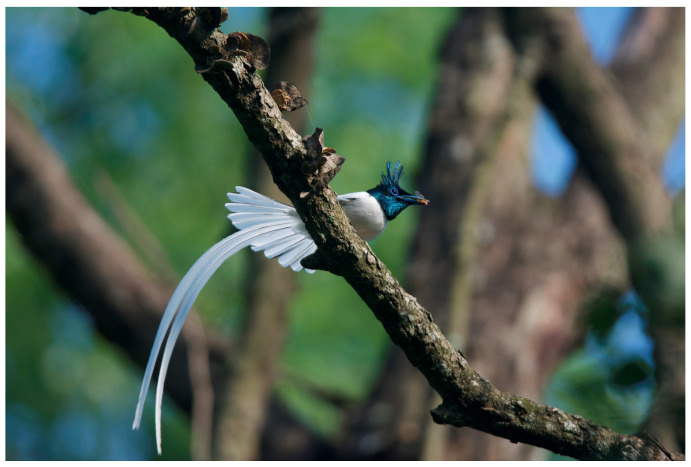
Male Himalayan Paradise Flycatcher (*Terpsiphone paradisi*). Chitwan National Park (henceforth CNP), Nepal. Photos by Nepal Dynamic Eco Tours, used with permission.

Game et al. (2013: 271) describe conservation as ‘far more complex’ than rocket science. Due to entangled concerns which include politics, caste, land-use, poverty, wildlife conflict, and the long-held belief that humankind inherently has the right to use wildlife and wild spaces, conservation has become a ‘wicked problem’ [13] with few clear solutions. These issues arise not only from the complex relationships between stakeholders but also from often polarizing ‘social, ecological, and economic elements’ which lurk beneath the surface of conservation dialogues [14]. As a global biodiversity hot spot (see Figure 2 and Figure 3), the small country of Nepal faces struggles with a growing number of these wicked conservation problems [15].

**Figure 4:**
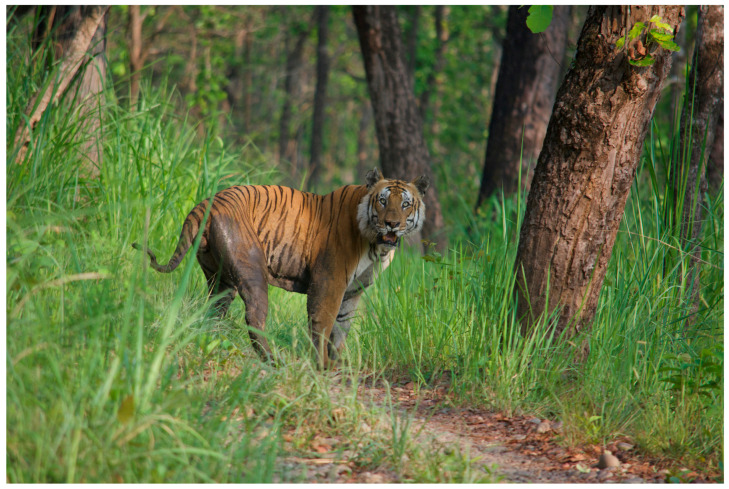
Wild Tiger, CNP, Nepal. Photo by Nepal Dynamic Eco Tours, used with permission.

**Figure 5:**
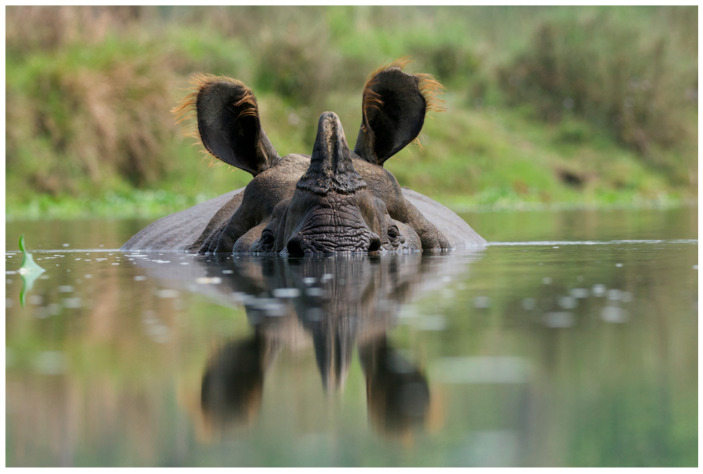
Rhino in lake, CNP, Nepal. Photo by Nepal Dynamic Eco Tours, used with permission.

For example, Nepal has designated more than a third of the country as protected area and has shifted its focus from single-species conservation to landscape-level efforts [16,17]. Nepal has also shifted from centralized governmental control of forestry and conservation programs to more locally representative forest users’ groups [16]. To combat illegal wildlife trade, Nepal has implemented greater interagency collaboration, increased enforcement of legislation, and formed anti-poaching teams (Acharya, 2016). Thanks to a combination of these efforts, Nepal has achieved several zero-poaching years for three of its charismatic flagship species: tigers (*Panthera tigris tigris*), elephants, and rhinos [18,19] (see Figure 4 and Figure 5). Nepal has also experienced increases in the numbers of wild rhinos and tigers, with rhino populations rising from fewer than 100 in 1966 to 649 in 2018 [20]. Likewise, tiger populations have more than doubled [17]. However, rebounding populations of these species cannot simply be described as conservation ‘successes’; instead, their overall effect on biodiversity preservation and impacts on larger multispecies communities should be considered.

**Figure 6:**
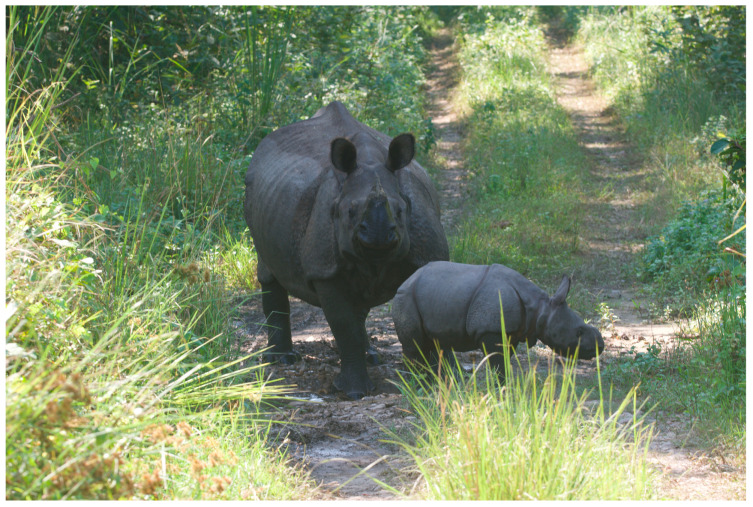
Wild rhino mother and calf, CNP, Nepal. Photo by Nepal Dynamic Eco Tours, used with permission.

**Figure 7:**
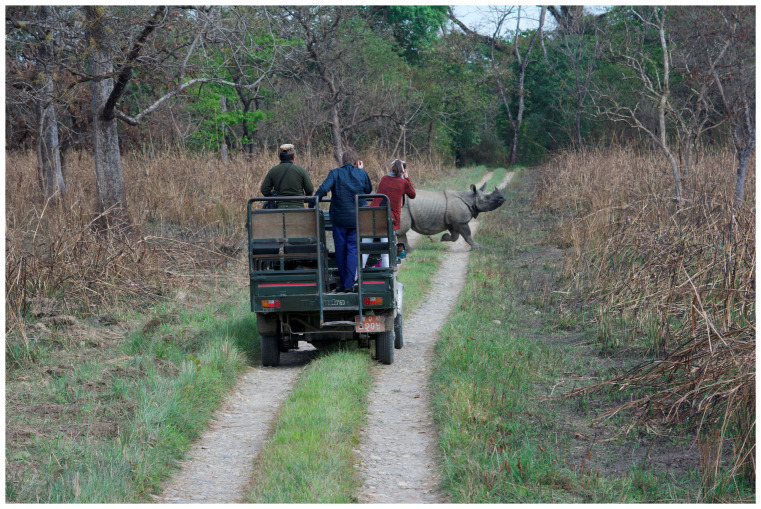
Tourists in jeep approaching a wild rhino, CNP, Nepal. Photo by Nepal Dynamic Eco Tours, used with permission.

Nepal’s conservation efforts have been laudable, and protected areas now represent a large portion of the country (as mentioned above) [17]. However, less than 2% of this protected land is contiguous, the rest is composed of fragmented habitats interrupted by human settlements [21]. Wildlife residing within these protected areas are still at risk from anthropogenic (human-derived) forces, including disease transmission, climate change, and competition for resources [17]. Furthermore, while wild individuals face fewer threats from poaching, they remain vulnerable to potentially harmful tourism practices [22,23]. For example, due to disturbances by tourist jeep safaris, many of Nepal’s protected species have become habituated to human noise, litter, and activity [24,25]. This habituation allows for jeeps to approach closer than recommended distances for human and other species’ safety [26,27] (see Figure 6 and Figure 7). When disturbances reach levels which are no longer tolerable, wildlife deserts traditional habitat, which in turn encourages tourism operators to venture deeper and deeper into protected areas to provide viewing opportunities for their clients [23,28]. Human incursions into these protected areas are extremely profitable, as entry fees provide the largest source of revenue for the national park [17,27]. For this reason, the government has been reluctant to further limit access to the national park despite decades of academic concern for the wild flora and fauna found within.

Another issue resulting from the ‘success’ of charismatic species conservation is that of human–wildlife conflict (HWC). Within protected areas and along their borders, human–rhino and human–tiger interactions are on the rise, as are other conflicts [29]. The proximity of agricultural lands and their associated water sources to protected areas means that ‘incursions’—a very anthropocentric word, which seems to imply human entitlement to land which previously served as wild animal habitat—by wildlife occur regularly [29]. Humans face financial and physical losses from these incursions, and wildlife are often killed in retaliation (see below). In addition, as wild populations rise, so do rhino deaths from territorial fights (there is not enough space left in the park, see below), falls into human-made wells, and fatalities from zoonotic (transferable to humans) or transmissible disease from livestock [25,30,31]. The increasing number of wild rhino deaths annually has left the government and researchers concerned that despite an estimated carrying capacity of 800–1000 rhinos [32,33], CNP may already have reached its limit.

A further problematic result of Nepal’s refocused conservation efforts was the forced relocation of indigenous populations to villages outside protected areas [17]. These relocations resulted in increased poverty and an inequitable proportion of HWC fatalities among already marginalized populations, such as indigenous Tharu people, Nepal’s oldest ethnic culture [34]. The removal of villages from the park has limited community income potential, thanks to loss of land, access to forest products for sale, and an inability to hunt. In fact, 88% of Tharu families stated they could no longer afford gas for cooking and had returned to a reliance upon firewood [34]. As the collection of forest products is now controlled by park management, families are forced to illegally enter protected areas to collect firewood, putting them at greater risk of death from tiger and rhino attacks [17]. Fatalities and financial losses also occur when villages relocated along the boundaries of protected areas fall prey to crop-grazing by wild rhinos and elephants [35,36]. These populations already faced inequitable representation in forest management and tourism operation ownership, and experienced fewer gains from tourism practices, placing them at greater risk of reliance on forest products for survival [34,37]. Increasing losses are intimately linked to the transformation of landscapes surrounding PAs from traditional habitat to highly desirable (for wildlife and humans) agricultural crops as former forest-dwelling human populations resettled outside the PAs [36,38]. Human population growth is further impacting land use practices, as demand for housing and agricultural land removes more and more wildlife habitat from areas surrounding the PAs [39].

## 3. Wild Elephants

**Figure 8:**
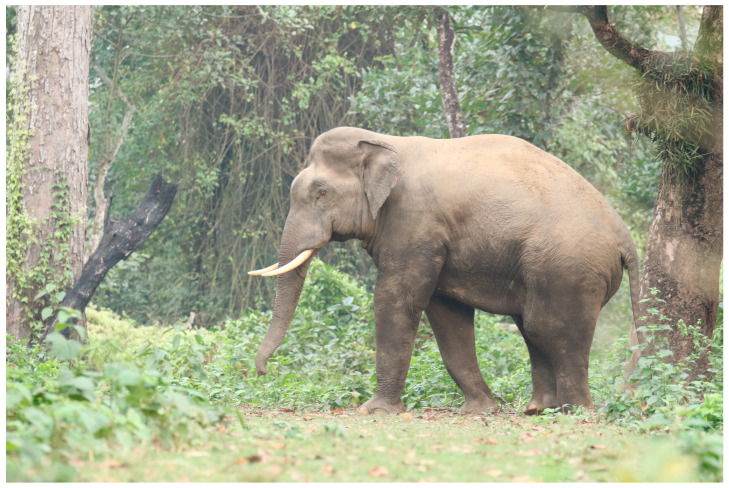
Wild bull elephant nicknamed ‘Govinda’, CNP. Photo by Nepal Dynamic Eco Tours, used with permission.

**Figure 9:**
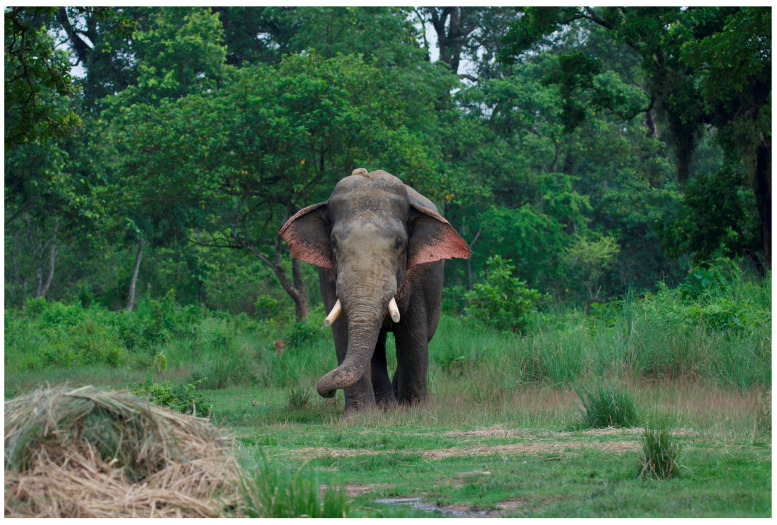
Wild bull elephant nicknamed ‘Renaldo’. Renaldo’s tusks have been cut for human protection. Photo by Ram Krishna Mahato for this project.

Like rhinos and tigers, Nepalese wild elephants face complex conservation problems (see Figure 8 and Figure 9). These wild individuals are in extremely short supply, with only 120–200 remaining among four geographically separated herds [3,40]. These herds are unconcerned with political boundaries, passing regularly back and forth across the Nepal/India border. These migration patterns, coupled with a lack of transboundary cooperation, make obtaining an accurate count difficult [41]. Even though so few remain, tourists list ‘seeing’ wild elephants as a primary motivation for visiting the protected areas of Nepal and the herds serve as a key selling point for both government agencies and tourism operators [35,42].

Unfortunately, Nepalese wild elephants likely represent a ‘doomed population’, defined as one which is unable to rebound in any meaningful way thanks to low numbers, lack of connected habitat, and the seemingly irreversible pace of habitat loss [43,44]. In fact, early documents recommended ignoring Nepal’s herds in favor of populations in other countries with a better chance of survival, or simply giving up and removing them from the wild completely [43]. However, more current research indicates that Nepal’s wild elephants represent an invaluable and unique source of genetic material, thanks to their continued geographic and social distance from other herds [45]. These elephants may, in fact, be key to both local and global conservation of the species [40,45].

The habitats of these wild elephants continue to be lost and fragmented thanks to human-derived causes, forcing herds into migration routes which place them ever closer to human settlements [40]. Narrowing routes lead to increased interspecies conflict and result in herds being viewed simultaneously as both incursive and endangered, valuable, and costly [7]. Elephant routes vary seasonally, meaning that herds regularly happen across villages and croplands [40,41]. Current crop-grazing (a term is used in place of ‘crop-raiding’, which is problematically value-laden, as these crops are planted within former elephant ranges) behaviour can be traced to herds’ natural movement patterns, rather than being contributable to the purposeful seeking out of cultivated plants (see Sukumar, 1990). Millet and rice, chosen by humans for their taste, nutritional value, and easy digestibility, likely attract passing elephants for the same reasons [41,46]. Crop-grazing behaviour may also be due to the higher sodium content found in cultivated crops—a mineral which is often low in wild elephants’ traditional diet [47]. This behaviour is considered an adaptation in response to declining access to, and variety of, plants within protected areas, and the lack of high-quality, high-nutrition food in their traditional habitat. These food sources are being lost to invasive plants (such as *Mikantha* spp., see Murphy, et al., 2013), competition with other wildlife and human agriculture, and livestock grazing [47]. In addition, some human crops simply taste good. Sugar cane, a popular crop in Nepal, is a known treat for elephants, and draws in passing herds. In addition, elephants can smell water kilometers away, and since water is readily available near agricultural lands, this garners the interest of migrating herds. Surprisingly, Pokharel, et al. (2018) found that elephants who engaged in crop-grazing had lower levels of certain stress hormones, a shocking discovery as elephants exposed to human activity, noise, and human-induced stress typically demonstrate higher levels of stress. They hypothesize that the higher caloric, protein, and general nutritional value of human crops, coupled with the relative ease of access to large quantities of food (rather than having to travel long distances for adequate plant material), instead increases the health and fitness of wild elephants, thus lowering their stress [47].

The ongoing expansion of villages into natural elephant habitat and habitat fragmentation and degradation have long been major contributors to crop-grazing behaviours and will remain a problem if human populations continue to grow at their current rate of 1.7% annually [48]. Crop-grazing contributes to human–elephant conflict, which has become one of the largest conservation issues in Asia and affects all 13 range states, costing millions of dollars annually [39,40]. These conflicts result in increasing numbers of injuries and fatalities to humans, and elephants face retaliatory killings over crop destruction, injury, or property loss [39,44].

The Nepalese government’s stated focus on preserving wild elephants has officials worried about the potential for escalating conflict as human populations increase. Between 2009 and 2020, elephants were responsible for 13% of all human–wildlife conflict in the Chitwan area (with 67 separate incidents of human–elephant conflict), and elephant–human conflicts demonstrated the highest potential for human fatalities [38]. Efforts such as unpalatable crop planting, trenching, noise makers, and fencing have been initiated in attempts to reduce the impacts of conflicts on humans [38,49]. The government has also increased financial compensation for loss in attempts to reduce HWC. However, in some areas, villages are deserting land due to extensive economic losses and property damage caused by elephants, and some families refuse to allow marriages into households which fall within areas of high elephant conflict [3]. As previously mentioned, forcibly removing indigenous people from protected areas has resulted in populations feeling they are less valued than wildlife, and these persons now face increasing risk from elephant–human conflict [50]. Relocating either elephant or human populations is a risky and unrealistic prospect, and unlikely to succeed given the ever-increasing numbers of humans and the declining availability of ‘natural’ habitat for translocating elephants (Menon and Tawari, 2019). Instead, elephant and human shared spaces need to be more equitably addressed in landscape-level plans which meet the needs of members from all socioeconomic tiers and species [39]. Novel approaches to solving this problem might include offering equal consideration to stakeholders from all involved species [51,52]. Viewing conservation simultaneously through the lenses of sustainability and social justice might also offer more balanced approaches.

## 4. Wicked Problem Two: Captive Elephants

Of course, conserving wild species may take a backseat to other, more pressing (at least to humans) needs. For example, Nepal’s human population faces ongoing social and financial challenges, with 40% of the population surviving on less than $1160 annually [53]. Six percent of the population is reliant upon the tourism industry for survival, and tourism represents ~7.5% of Nepal’s GDP [54]. Thanks to the purported (yet controversial) benefits offered by tourism practice (see Bookbinder, et al., 1998; Nyaupane and Poudel, 2011; Puri, 2019), the Nepalese government decided to link poverty reduction with conservation efforts and continues to heavily market nature-based tourism [9]. This marketing works, with 60% of international tourists (430,000 people in 2018) visiting the fragile and biodiverse protected areas of Nepal annually [42]. However, this mass tourism makes it difficult to balance the purported goals of protecting wildlife and wild spaces with their value as commodities [16,55].

**Figure 10:**
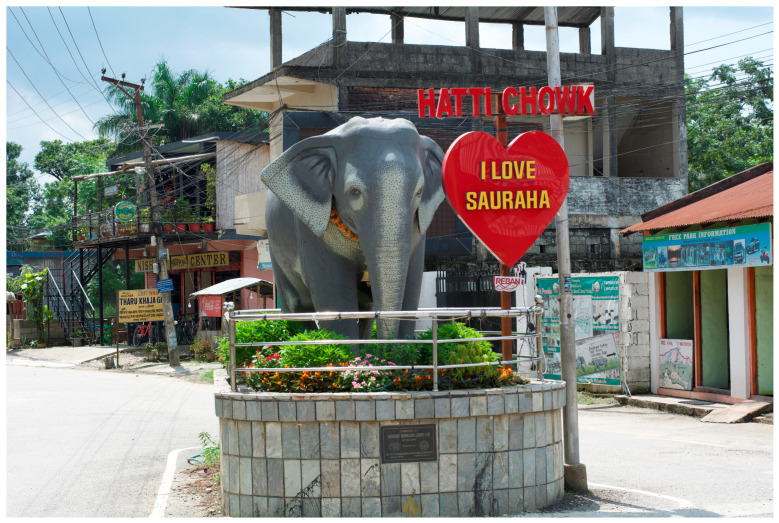
Hatti Chowk (elephant statue public gathering spot). Photo by Ram Krishna Mahato for this project. Used with permission.

Chitwan National Park (CNP) is the busiest protected area in Nepal and has been described as ‘the last surviving example of the natural ecosystems of the Terai’ [17]. CNP is also home to several wild bull elephants and one of Nepal’s four remaining matriarchal herds. It is in the tourist-laden towns surrounding the park where one finds another kind of endangered elephant—those used for tourist safari transportation. These elephant-backed safari rides bring in the second highest amount of revenue for the area [27], and the elephants serving as the vehicles for these adventures live a very different life than those of their governmentally held or wild cousins. While they are members of the same endangered species, privately-owned elephants face increased challenges to their health and welfare [7,30,56]. Unlike governmentally held elephants, privately-owned individuals lack any legal protections regarding their treatment, nutrition, or usage (see Figure 10).

Before the COVID-19 pandemic, 52 of these privately owned individuals worked in the Chitwan area. These elephants carry up to six tourists at a time on a howdah (riding platform). Each ride lasts approximately 90 min, and each elephant may perform up to nine rides a day through the jungle [57]. These elephants are massively expensive, costing approximately 90 times more than the average annual worker’s salary, meaning that only those of higher socioeconomic standing can obtain them [58]. They represent a huge financial opportunity, and a huge financial burden thanks to their upkeep costs, which may run up to $19,000 USD annually (PC, 2021) [57]. These tourism elephants are caught in a liminal space—seen in Buddhist and Hindu literature and society as divine creatures, as literal vehicles for tourism practice, as symbols of power and wealth, but also as disposable commodities which can be sent off when no longer ‘useful’ [7,59]. They serve as symbols for international organizations hoping to bring awareness to issues facing elephants in captivity, and as representatives of Nepalese history and culture [60,61]. Working in the hattisars of Nepal is challenging; thanks to my interest in elephant owners and traditions, I initially faced alienation by advocacy organizations hesitant to share their goals lest the owners limit access to elephants. Then, when I began researching advocates and NGOs, elephant owners became hesitant to share their stories, worried that I was a spy for those who might want to see elephant-based tourism end.

Not only are researchers caught between these groups, but so are those who spend the most time with elephants, their mahouts. While elephant caregivers have long been members of marginalized communities [4], at least in the past their choice of profession might have resulted in stable employment with the government or allowed them the use of elephants for farming and logging [62,63]. These elephant caregivers have traditionally come from indigenous tribes and/or low-caste families; they are uneducated, poorly paid, and overworked [64]. They are alternatingly viewed by the community as the keepers of traditional knowledge and ostracized as violent men with gambling or drinking problems [4,34,64].

Like many southeast Asian countries, the mahout–elephant relationship within Nepal has undergone changes over the past five decades [5,65]. For example, the long-term bonds which mahouts once shared with their elephants have, in many cases, been replaced with unstable or temporary working relationships [5,56,66]. In many cases, these relationships have become damaged or neglected as inexperienced mahouts with little training have replaced older mahouts who grew up in hattisars (see also Szydlowski, 2024). The position of mahout as a familial job is no longer the norm (with some exceptions, see Mumby 2019), and it is now young men with little embodied knowledge of elephants who are left to care for these captive endangered beings. The changing skill level of caregivers, the loss of strong mahout–elephant bonds, and the loss of traditional and embodied knowledge places mahouts and elephants at greater risk of neglect, injury, or death [64,67,68].

**Figure 11:**
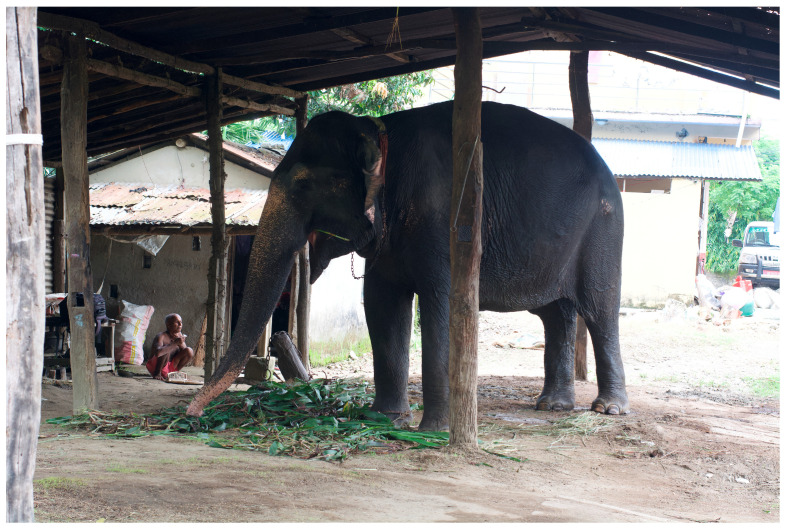
Typical elephant *hattisar* (stable) in Sauraha, Nepal, showing chaining methods. Photos by Ram Krishna Mahato for this project, used with permission.

**Figure 12:**
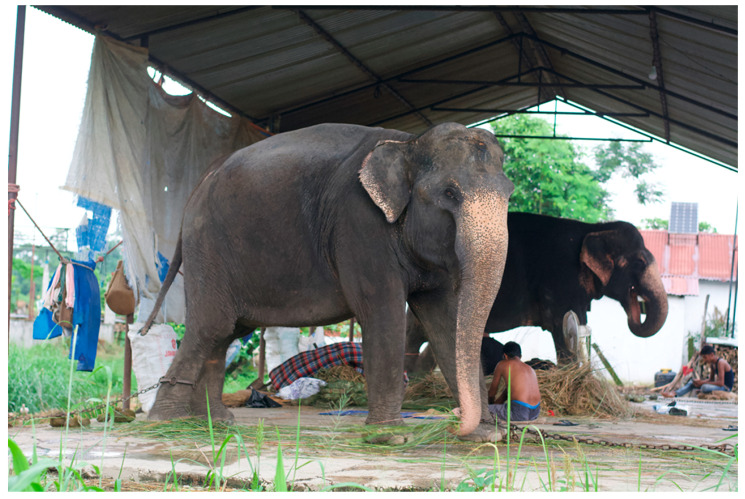
Another typical elephant *hattisar* (stables) in Sauraha, Nepal, showing chaining by front and back legs. Photos by Ram Krishna Mahato for this project, used with permission.

**Figure 13:**
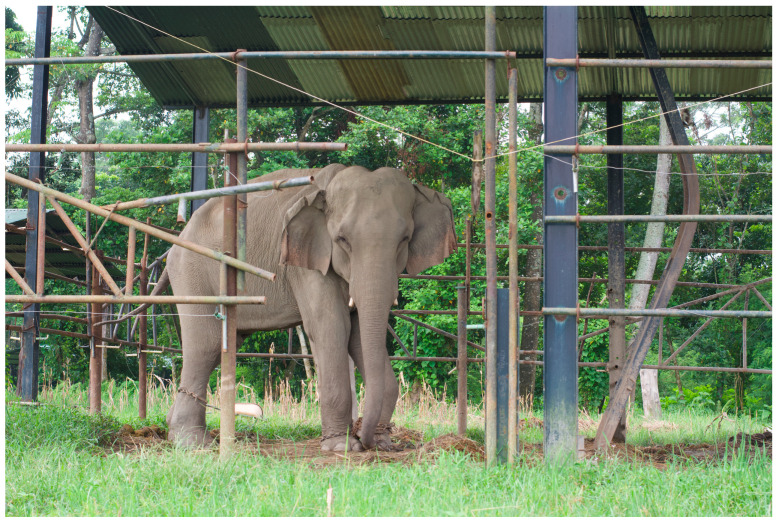
The only adult male elephant in Sauraha who is privately owned. Kumroj, Nepal. Photo by Ram Krishna Mahato for this project.

Within Nepal’s hattisars, I have witnessed horrific abuse, malnutrition, and neglect, but I have also seen deep and tender love. These seemingly contradictory human treatments of elephants, and the fact that numerous international organizations attempt to influence the management of elephants in Nepal, are major reasons that the ‘elephant situation’ within the country is so complex [7] (see Figure 11, Figure 12 and Figure 13 for an example of Nepalese stables). The influence of both local and international organizations, and the interplay of this influence with changing societal perspectives on the practice of captive-elephant management in Nepal, is beyond the scope of this article but is an area of ongoing study for the author [7,30].

## 5. Additional Challenges

Captive elephants throughout South Asia suffer from a lack of veterinary care as well as low standards for their management, handling, and husbandry, and Nepal is no different [39]. However, Nepal’s elephants face vastly different situations than those in the villages of Laos, the camps of Thailand or Myanmar, or the temples and streets of India. There have been studies within other Asian countries demonstrating a range of successful human–elephant relationships, a historical acceptance of elephants as community members, and even positive health and welfare implications of living in certain camp situations (for example, see Bansiddhi, et al., 2020; Lainé, 2019; Lehnhardt and Galloway, 2008). However, given the small number of both wild and captive elephants residing in Nepal, until recently, consideration of their welfare has largely escaped the interest of the international research community (see Szydlowski, 2024). In addition, the housing, nutrition, and management styles used within Nepal vary greatly compared to those seen elsewhere in Asia, making assessing elephant welfare even more difficult.

Another challenge arises from the use of elephant breaking rituals in Nepal. While these rituals, such as the ‘crush’, pha jaan, or hattiko talim [62,69], have purportedly fallen out of favor in several other Asian countries thanks to international pressures, they remain the standard way of creating ‘usable’ elephants within Nepal [57,62,70]. This ‘desensitization’ process, which involves both gentle care (such as singing and rubbing) and violence (fire, abuse, and stabbing), often results in long-lasting trauma to both mother and calf [66,71]. This type of breaking process may also result in injuries or fatalities for mahouts, when formerly ‘broken’ elephants later attempt to dominate their handlers [71,72].

Rather than living in camp situations as they might in other parts of Asia, elephants in Nepal live singly in small stables behind businesses. These elephants lack any tactile, olfactory, or visual contact with conspecifics, despite the wealth of data documenting the importance of these social connections (see EAZA, 2020; Poole and Granli, 2008; Prado-Oviedo, et al., 2016). In addition, the food provided to captive elephants in Nepal differs greatly from that of wild herds or captives in other Asian countries. As browsers, wild elephants typically spend up to 60% of each day foraging on a large variety of plant species, and camp elephants in other countries often graze throughout their day [73]. Captive elephants in Nepal, however, rely upon domesticated crops or provisioned foods (such as rice, which is not a natural food source for wild elephants in Nepal) (PC, 2017, 2019). These provisioned foods are fed at limited sessions several times a day, and often require the use of threats to get elephants to consume them (PC, 2019, 2022). As one interlocuter stated, ‘They are given food they don’t like and then beaten when they won’t eat it’. In addition, this provisioned food does not allow for typical behaviour during consumption, so while it may fulfil physical needs, it likely does not fulfil mental welfare needs [74]. For example, wild elephants prepare their food in several ways prior to consuming it, such as dropping, breaking, and bending it [75], allowing for further mental and physical exercise opportunities as they manipulate food items. Limiting time for captive elephants to manipulate and consume their food has been shown to cause increases in stereotypic behaviours, changes in body condition scores, decreased welfare, and negatively impacts digestive health [5,72].

Studies of health and welfare among Nepalese captive elephants found that the majority suffered a variety of physical ailments ranging from wounds and abscesses to malnutrition, inappropriate sleeping conditions, broken nails, joint issues, vaginal prolapse, tuberculosis, and other diseases [7,56,76,77]. In addition, these elephants exhibited mental health issues such as maladaptive passivity, stress, stereotypies, loneliness, and other behaviours which may be linked to early separation from their maternal herd or ongoing social isolation.

For example, when not on safari (or during the off season) elephants spend most of their day chained in one position and this inability to move around for exercise or to avoid soiled areas can be detrimental to both physical and mental health [77,78,79]. Studies have linked bacterial causes of arthritis to unclean conditions or unsuitable substrates, indicating that poor husbandry decreases foot and joint health [80]. The stress caused by resultant arthritis may further impact foot health, creating circular issues [72,80]. In addition, the current chaining method (one front and one back foot using short chains) changes the standing position of these elephants, which further negatively impacts their joint health [57,74,76].

Welfare and health issues are a point of contention between various stakeholders and external agencies involved in captive elephant management within Nepal. At this juncture I would like to note that these privately-owned, captive elephants are (with a few exceptions) illegally obtained. Nepal has been a signatory to CITES since the 1970s, and as such is prohibited from protected animal trade across national borders for commercial purposes [81]. However, removing elephants from the wild has also been prohibited in Nepal since the 1970s, meaning that private owners wishing to continue garnering income from safari must import individuals from India [2] (PC, 2019). These individuals were walked or trucked across the border, and while owners openly acknowledged their illegal activities, officials denied any knowledge of these transfers [7,82]. Even with protective regulations in place, government documents have been quick to point out exactly which Indian cattle fairs potential investors should visit if they would like to purchase elephants for use in the tourist trade [3].

In the fall of 2021, Nepal’s supreme court committed to the enforcement of CITES (The Convention for International Trade in Endangered Species) regulations and a cessation of elephant passage across the India–Nepal border. In addition, the government required the registration of all existing privately held elephants (this process is still underway). Unregistered elephants were to be confiscated, although it remains unclear who is responsible for exercising this option; the government, the elephant owners’ association, and the NTNC all denied responsibility. Despite issues with implementation, the government’s commitment to stopping illegal trade and keeping current elephant residents within the community is a positive step towards affirming the important connections between Nepali culture, history, and transspecies relationships.

In the past six months, mahouts and elephants have been turned back as they attempted to cross into India [83]. However, this new enforcement of legislations is not without danger for elephants. One female, illegally sold by a Nepalese businessman to an Indian buyer, was being transported across the border when reported to authorities by a Nepalese animal advocacy organization. This elephant, Kajol Kali (female elephants are surnamed ‘Kali’ and males ‘Gaj’ in honor of Hindu gods), was returned to Sauraha, but without a local owner or stable she had few options. She was severely malnourished, having been traveling for multiple days and kept hidden without adequate food as her owners tried to ‘sneak’ her across the border. The NTNC (see introduction) and national park staff claimed they were uncomfortable getting involved in disputes involving private owners. However, it is the NTNC’s veterinary staff which is responsible for the health care of all privately held elephants in the Chitwan area, thanks to a long-standing agreement with the owners’ cooperative [7].

Despite the supreme court requirement that illegally sold elephants be confiscated, no entity reportedly felt they had the authority to enforce the new ruling, and Kajol continued to starve. Local and NTNC veterinary staff were aware of the situation (having been invited to visit the facility by several stakeholders) and refused to examine the elephant. The NTNC veterinary staff did finally offer slight supportive care, after six months. NGOs paid for the necessary supplies and placed pressure on the NTNC. Another local elephant owner offered the use of his stable, and various I/NGOs stepped in to assist with costs associated with feeding and care. One sent staff daily to help ease the burden of the single mahout caring for Kajol (most elephants are assigned two mahouts). Despite the mobilization of NGOs, individuals, and financial support, Kajol did not recover from her malnutrition and fell numerous times, perishing after 8 months of struggle. This case highlights the liminal nature of privately-owned captive elephants in Nepal. With little oversight and no legislation regarding their care, captive elephants remain liminal beings. They are caught between human desires: the desire to see or be near these embodiments of gods, to use them as transport for viewing their wild cousins, to maintain ‘traditional’ multispecies relationships, and to garner income at levels inaccessible for most other community members. Yet, as will be discussed next, the health and welfare of these captive individuals is likely key to the survival of wild populations.

## 6. Wicked Problem Three: The Captive–Wild Interface

Captive individuals impact human safety by providing anti-poaching patrols and can help run off wild elephants in populated areas [17]. In addition, they provide employment for a marginalized population, mahouts, and (purportedly, see previous sections) bring in important tourism revenue for the towns surrounding CNP. However, these activities also increase the risk to populations of wild elephants and other wildlife. The health and welfare of captive individuals is therefore important for reasons that go beyond concerns for the maintenance of the tourism industry, their right to be treated as intrinsically valuable living beings, or the desire of welfare organizations to see an improvement in conditions.

**Figure 14:**
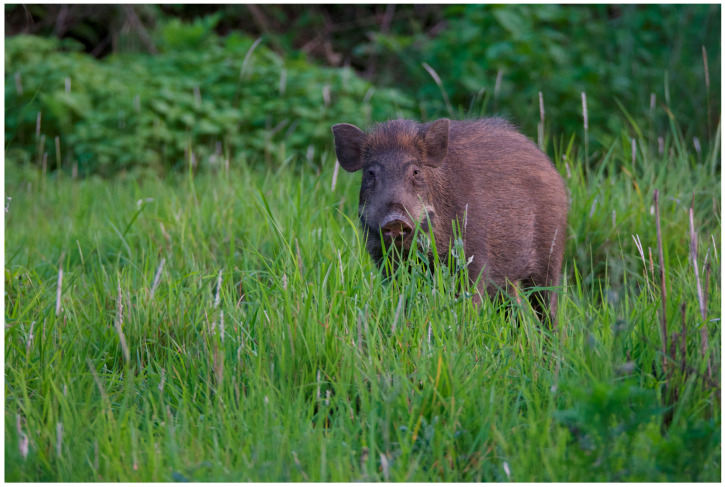
Wild boar CNP, Nepal. Photo by Nepal Dynamic Eco Tours, used with permission.

For example, captive elephants residing in private hattisars share their spaces with domesticated livestock as well as local wildlife such as wild boar (*Sus scrofa,* see Figure 14, four species of deer, rhinos, and many smaller species. These elephants travel through protected areas on the way to, and during, tourist safaris each day. Approximately 23% of these captive individuals carry tuberculosis, and others carry elephant endotheliotropic herpes viruses [84]. Other diseases such as rabies, foot and mouth disease, respiratory infections, tetanus, salmonellosis, and internal or external parasites have all been reported in Nepalese elephants [2,76]. Concerns regarding the passage of these diseases into wild populations have become a reality, with fatalities among wild elephants and greater one-horned rhinos increasing [31,85].

The management of these captive populations of elephants, therefore, is vitally important to the health and welfare of wild elephants [80,85]. Interactions between wild bulls and captive females during mating and overlapping natural foraging areas, for example, create opportunities for the passage of disease into wild populations, puts a strain on forest resources, and may impact the survival of wild populations [2,64]. There is a pressing need for further research which considers the health and welfare of captive individuals as an important part of overall conservation efforts and one world, one health type initiatives.

Thanks to Nepal’s reliance on tourism for the survival of many marginalized communities, and the income generated for owners by elephant-backed safari, it is unlikely that elephant tourism will simply disappear from Sauraha. In fact, owners and veterinary staff expressed concern that if elephant safari ended, tourism would follow, and thus are very interested in seeing it continue. What is needed, then, are alternatives which consider the needs of humans, elephants, and other species living in or around the area. One such approach is to identify ways in which disparate entities, such as owners, advocates, I/NGOs, and local communities can work together to ensure continued income from tourism while ensuring elephants are well cared for and healthy.

## 7. A Novel Approach to Conflict Resolution

Allow me to return to the introduction to this presentation, and my comments on my own naivety. As I undertook my survey of the stables of Nepal, I began to seek out local, national, and international organizations who purported a desire to ‘help’ captive elephants. These organizations ranged from the quasi-governmental NTNC and the Chitwan Nature Guide Association, to the Elephant Owners’ Cooperative and several international interests. Some of these I/NGOs have a permanent presence in Nepal, while the majority rely upon locals to care for their elephants and distribute funds.

Each of these organizations purports to ‘help’ elephants and profess a sense of responsibility to the captive elephants near Chitwan. The problem with undertaking the ‘rescue’ of these elephants lies in the lack of knowledge of local elephant–human relationships, Nepal, elephant health, mahout culture, and so on. Most of the people who became involved in these NGOs did so after being moved by an experience with an individual elephant (see Lorimer, 2009; 2010). While some went on to involve experienced staff or local people in their projects, others simply started to fundraise, post on social media, and hope that the future would bring elephants to their facility. Still others had no facility, no plans, and no relationship with elephants beyond their desire to help. Rather than communicating with other NGOs, elephant owners, or the government, they simply began to fundraise using photos of elephants in adverse conditions [7].

During my time in Nepal, I realized that while these agencies were aware of one another, few had any type of contact with one another. In addition, most INGOs attempting to improve elephant welfare were avoiding the owners of said elephants (for a variety of reasons; some viewed owners as ‘mafiosos’ or ‘evil men’, some practiced simple avoidance to avoid conflict), and ignoring the NTNC’s veterinary staff—the very people who knew mahouts and their elephants best. As I bemoaned this lack of communication during a meeting with the NTNC’s veterinarian, he somewhat sarcastically proposed an unusual idea. Would it not make a great addition to my research, he suggested, if I could bring all these entities together for a meeting? While he was joking, the idea continued to nag at me. I decided that thanks to my decades of work in Nepal, and my long efforts to gain the trust of elephant owners, NGOs, grassroots organizations, and community members, I was in a unique position to extend an invitation to what I jokingly entitled the first ‘Sanctuary Summit of Nepal’. Sauraha is a small town, and I hypothesized that by getting individuals acquainted to the point where they might say hello when inevitably passing on the street, these groups might find common ground in their efforts to improve elephant welfare. I composed and sent an email inviting nature guides, INGOS, local community leaders, welfare groups, owners, ex-pats, and veterinary staff to coffee at a local shop. The initial response was encouraging, with owners and activists alike agreeing to come…if I kept them informed as to who else was invited.

The morning of the summit, I sent reminder emails and made calls to encourage attendance. When NTNC staff, members of an INGO, a visiting veterinarian, and a local hotelier arrived, I was thrilled. While these guests were aware of each other, few were formally acquainted. Introductions were made, and they began to chat about their hopes, needs, and expectations. INGOs and veterinary staff agreed to work together on communication with owners and one another. As we continued to wait, they informed me that it was unlikely any elephant owners would arrive. Rather than refusing my invitation directly and risk disappointing me, they explained that it is more culturally acceptable to simply not show up. In the end, my own cultural expectations of attendance at agreed events set me up for potential disappointment. But only for a few moments, as I soon realized that supporting any opportunities for connecting entities active within Nepal had the potential to ripple into larger collaborations.

Following the summit, other invited organizations began an email chain to summit invitees. These emails continue sporadically, offering occasional updates on organizations and encouraging dialogue with the larger group of elephant advocates. In addition, these email exchanges proved useful in clarifying each organization’s definition of ‘sanctuary’, and ‘care’, and providing insight into the ethical norms and best practices of each. While the initial number of physical participants was low, the summit proved helpful in facilitating communication among stakeholders in Nepal and continues to provide a link between formerly disparate entities. While I did not realize it at the time, I was using wicked-problem-solving skills. As Mason et al. (2018) suggested, improving interactions among stakeholders may allow for more effective conservation conflict management. Among the suggestions for improving conservation outcomes are decentralizing decision making, bringing in diverse sources of knowledge, being transparent about failures, and focusing on outcomes rather than strategies [14]. Though the summit may have arisen out of a naïve attempt to get others to view elephant care through a more inclusive lens, it was successful in opening a dialogue between interested parties which then encouraged further communication.

Other organizations, however, continue to work alone, often without the permission of elephant owners. These organizations trespass upon private land to make improvements to stables or offer supplemental food for elephants and mahouts. Several focus simply on raising funds for generic elephant ‘support’ (without financial reports, oversight, boards of directors, etc., there is little hope of tracking where exactly this support goes). Some of these efforts have, according to mahouts and organizational staff, undoubtedly improved the welfare of individual elephants. However, the lack of organizational cooperation, or cooperation with hattisar owners, is problematic. It promotes adversarial relationships between those agencies including elephant owners in the conversation, potential funders of projects, mahouts, and those respecting the ownership rights of property (stables) [86,87]. It also perpetuates neocolonial attitudes, that only outsiders could possibly know ‘what is best’ for elephants [87,88] (see also Liu and Leung, 2019). It also places mahouts, an already marginalized community, in a position between advocates and owners. While they want to improve their stables to improve the health of their elephant co-workers, they also rely heavily on their jobs to survive a difficult economy. Many report that being a mahout is one of the last remaining jobs for undereducated communities, and worry that without these jobs, their families would not survive.

Lastly, the involvement of INGOs who do not include local advocates, governmentally appointed agencies, or local organizations in the planning process thus limits the ability of these local groups to promote societal change [86,87]. Rather than natural shifts in perspective on the use of animals arising from within the community, owners reported feeling that they are being pressured from exclusively outside interests. Owners report that they are responding to this pressure by disallowing outsider involvement with ‘their’ elephants, stables, or employees, and doubling down on their commitment to offer elephant-backed safari numerous times a day [7].

## 8. Conclusions

Rather than seeking a single answer based on conventional approaches, Game et al. (2013) suggest that conservationists should instead incorporate innovative, flexible, adaptive, creative, and diverse methods in their practices. Anthropological, and especially anthrozoological, methodologies and practices may offer novel and interdisciplinary approaches to the wicked problems of conservation.

**Figure 15:**
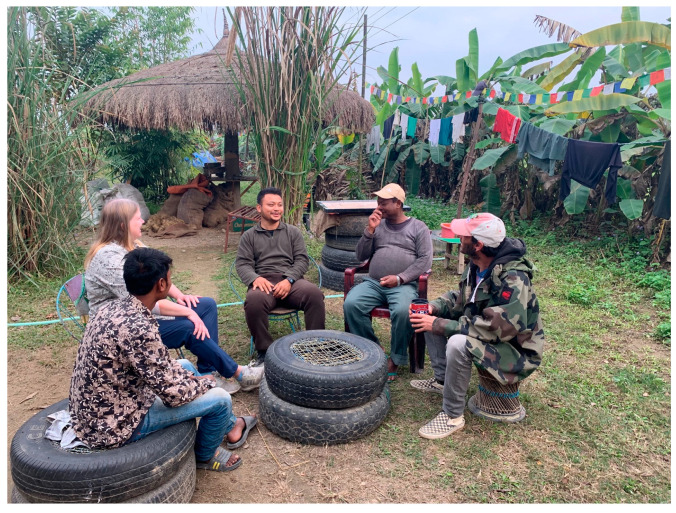
The author meeting with mahouts near Kumroj, Nepal. Photo by Stand Up 4 Elephants, used with permission.

One such novel approach is to ensure that all stakeholders, including those of multiple species, are offered equal consideration in conservation efforts. To that end, Nepal has shifted from single-species efforts to landscape conservation programs. What is needed next is a shift to include consideration of the agency and the desires of undomesticated species held in captivity, such as endangered elephants. Additionally, both governmental and nongovernmental organizations should focus on supporting mahouts and other marginalized communities which are reliant upon both tourism and conservation efforts, and upon whom captive species rely (see Figure 15). Rather than vilifying owners or typecasting mahouts, I/NGOs and advocates interested in improving elephant living conditions should continue to promote interagency relationships and those with elephant owners and co-workers. As the recognized owners and managers of these elephants, the decision to improve elephant management practices will ultimately fall to them. Creating opportunities for alternatives to safari income might demonstrate that ceasing elephant-backed safari is available, and NGOs might be well served to focus on solutions which equally support mahouts, elephants, and owners. It is also important to consider that while positive bonds between elephants and humans should be supported and celebrated in the hopes of improving the daily lives of both species, perhaps reconsideration is needed of the ethical issues surrounding the keeping of social and intelligent species in captivity.

Multiple other issues surround elephants in Nepal: the passage of disease between wild and captive populations, the reliance on wild bulls for captive reproduction (meaning wild males regularly enter captive stables), and the danger to both humans and elephants which arises from co-work and co-living. These issues require further examination to identify ways in which traditional practices, beliefs, and income sources can be maintained, while adjusting to changing human perspectives on the use of animals for exclusively entertainment purposes such as tourism. Anthrozoology, thanks to its interdisciplinary nature, may prove an ideal discipline for such an examination.

**Figure 16:**
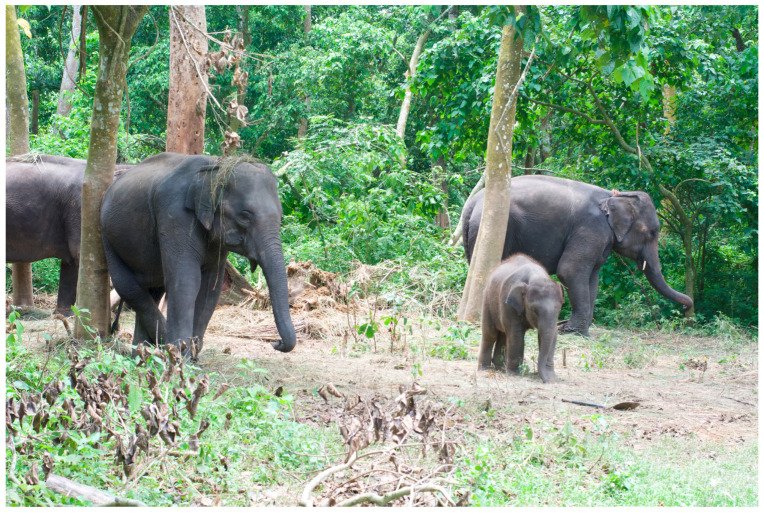
A privately owned, chain-free matriarchal herd in Kumroj, Nepal. Photo by Ram Krishna Mahato for this project.

Likewise, the issues facing wild populations, such as distance between matriarchal herds and wild males, too few individuals to ‘save the species’ in the wild, and increasing HWC, need to be addressed through the lens of environmental justice. Perhaps a novel solution which embraces the needs of both captive and wild populations, as well as humans, can be found. Allowing captive elephants to experience a semi-free-ranging lifestyle has allowed for improvements in elephant health and welfare, along with fewer mahout injuries in other countries, and might be worth further examination in Nepal. Likewise, a semi-free-ranging lifestyle would increase opportunities for expression of agency, choice of mates and subsequent increase in reproduction (although the ethical implications of breeding more elephants for captivity is fraught and requires further reflexivity), ability to access a wider variety of plant material with less work by mahouts and would allow the opportunity for elephants to engage in social interactions with conspecifics. This semi-free-ranging lifestyle may also eventually allow for the eventual release of captive individuals into wild herds, which was attempted in the past and has been successful in other countries (see Kharel, 2002; Varma and Ganhuly, 2011). One elephant owner has been experimenting with this method near CNP, see Figure 16 (a discussion of the experiment can be found in Szydlowski, 2023b [89]). Release efforts, however, can only succeed if captive individuals are free of communicable disease, are in optimal health, have built herd relationships, and have experience with non-provisioned fodder. This solution would require extensive cooperation and planning involving all stakeholders. Therefore, it may be beyond reach in the foreseeable future, and focus should be instead placed on providing legislation which outlines welfare standards for captive individuals, and the placement of females in small, social groups in chain-free facilities (See previous section).

Another novel approach might include embracing Scheper-Hughes (1995) concept of advocacy, in which researchers are encouraged to become involved. As individuals who ‘know’ their research subjects and are ‘morally engaged’ in their lives, researchers are in a unique position to offer insight into the perspectives of multiple stakeholders [90]. Choosing not to be involved is a morally indefensible position, according to Scheper-Hughes (1995), as it sets the researcher above their participants and their associated life events. Researcher advocacy of course requires a great deal of reflexivity to ensure that neocolonial attitudes are not being perpetuated and that power relations do not favor the researcher but rather offer equal support to all participants. Approaching the conservation of wild and captive individuals from an advocacy standpoint also allows for an inclusion of environmental and social justice perspectives.

It is likely that the novel solutions needed for the wicked conservation problems outlined here lies in a combination of ideas. What is clear is that singularly focused approaches have been unsuccessful, and there is a pressing need to consider not only the wide variety of human stakeholders involved in conservation efforts, but also members of those species being ‘conserved’.

## Data Availability

Data has not been made publicly accessible due to safety concerns for human and elephant participants. Further inquiries can be directed to the author.
